# Successful Management of Acute Subdural Hematoma in Deep Brain Stimulation Patient: A Case Report and Literature Review

**DOI:** 10.7759/cureus.61469

**Published:** 2024-05-31

**Authors:** Tatsuya Tanaka, Huynh Ngoc Yen Nhi, May Pyae Kyaw, Yusuke Otoki, Yukinori Takase, Kiku Uwatoko, Hiromu Minagawa, Motohiro Yukitake, Takashi Agari, Eiichi Suehiro, Tatsuya Abe, Akira Matsuno

**Affiliations:** 1 Neurosurgery, International University of Health and Welfare Narita Hospital, Narita, JPN; 2 Neurosurgery, Kouhoukai Takagi Hospital, Okawa, JPN; 3 Neurology, Kouhoukai Takagi Hospital, Okawa, JPN; 4 Neurosurgery, Saga University, Saga, JPN

**Keywords:** traumatic brain injury, intracranial pressure, minimally invasive, small craniotomy, surgical technique, endoscopic hematoma evacuation, acute subdural hematoma, parkinson disease, deep brain stimulation

## Abstract

Deep brain stimulation (DBS) has emerged as an important therapeutic option for several movement disorders; however, the management of acute complications, such as acute subdural hematoma (ASDH), remains challenging.

This is the case of a 71-year-old woman with Parkinson’s disease who developed ASDH 12 years after bilateral DBS placement. On admission with altered consciousness, imaging revealed significant displacement of the DBS electrodes because of the hematoma. Emergent craniotomy with endoscopic evacuation was performed with preservation of the DBS system. Postoperatively, complete evacuation of the hematoma was confirmed, and the patient experienced significant clinical improvement.

ASDH causes significant electrode displacement in patients undergoing DBS. After hematoma evacuation, the electrodes were observed to return to their proper position, and the patient exhibited a favorable clinical response to stimulation. To preserve the DBS electrodes, endoscopic hematoma evacuation via a small craniotomy may be useful.

## Introduction

Deep brain stimulation (DBS) has gained considerable acceptance as a highly effective treatment for medically refractory movement disorders, including Parkinson’s disease, dystonia, tremors, epilepsy, and certain neuropsychiatric conditions. Complications include intracranial hematoma, infection, and hardware failure. The causes of subdural hematoma (SDH) in patients undergoing DBS are intraoperative surgical procedures or traumatic brain injury [1-11]. The management of complications, such as DBS electrode displacement due to hematoma and hematoma removal, remains a challenge.

We report a case of acute SDH (ASDH) 12 years after bilateral DBS placement. The DBS system was preserved during hematoma evacuation, and the patient benefited from stimulation therapy despite significant electrode displacement.

## Case presentation

**Figure 1:**
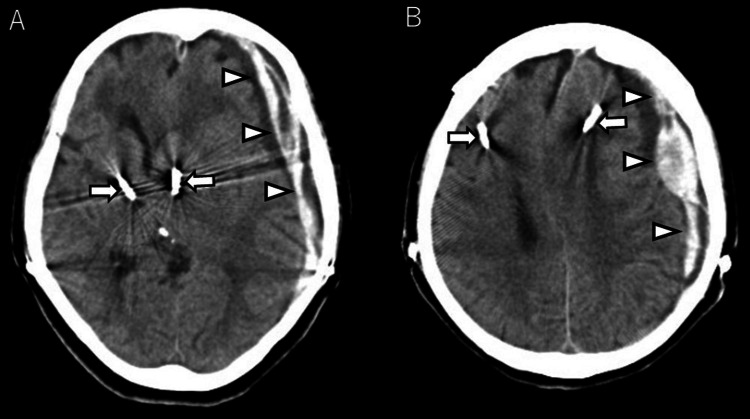
Initial non-contrast head computed tomography (CT) (A, B) Initial non-contrast head computed tomography showing a thick acute subdural hematoma (arrowhead) and midline shift and significant displacement of the deep brain stimulation electrodes (arrow) with the overall brain shift.

A 71-year-old woman presented 19 years ago with intermittent tremor in her right hand. Due to the progression of symptoms and poor response to medication, she underwent placement of bilateral subthalamic nucleus DBS electrodes 12 years ago. Her gait disorder gradually worsened and she had recently fallen several times. She presented to our hospital with altered consciousness at home. The patient’s medical history included Parkinson’s disease, pelvic fracture, rib fracture, and sacral perineural cyst. The history of this injury is unknown. The patient was not taking any antiplatelet or anticoagulant medications. Upon presentation, the patient’s Glasgow Coma Scale (GCS) score was 7 points. Initial non-contrast head computed tomography (CT) showed left ASDH with midline shift and marked displacement of the DBS electrodes with the overall brain shift (Figure 1).

Therefore, an emergent craniotomy with endoscopic evacuation was performed under general anesthesia. The DBS system was kept on, and no monopolar was used during the surgery. A linear skin incision of approximately 8 cm was made parallel to the DBS lead, and a small craniotomy of 5-6 cm in diameter was performed to preserve the existing stimulator leads, including the DBS burr hole and the locking mechanism. After a cruciate dural incision, the hematoma was removed under direct vision and then under endoscopic vision with the suction cannula. A drainage tube and an intracranial pressure sensor were placed in the subdural space.

**Figure 2:**
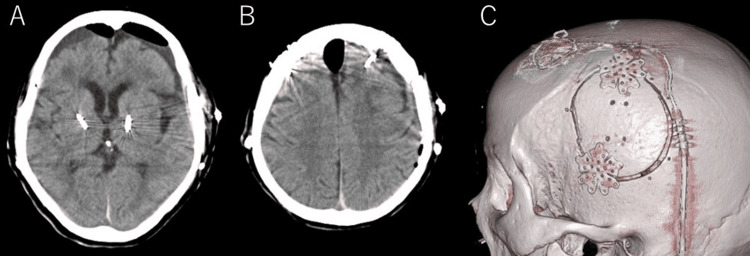
Postoperative head computed tomography (CT) and three-dimensional CT (3DCT) (A, B) Postoperative head CT showing sufficient hematoma removal and improved midline shift after the procedure. (C) 3DCT showing the cranial bone after the procedure. A linear skin incision was made parallel to the deep brain stimulation lead, and a small craniotomy was performed.

Postoperative non-contrast CT revealed complete removal of the hematoma, and the DBS electrodes seemed to shift back in place (Figure 2).

No abnormalities were observed in the postoperative impedance measurement of the DBS device. The patient was alert postoperatively. Her modified Rankin Scale (mRS) score was 3 at discharge on postoperative day 29. Six months following the surgical procedure, no recurrence was observed with an mRS score of 3.

## Discussion

Hematoma evacuation for ASDH in patients undergoing DBS is rare. Reviewing the literature, only six cases, including our case, have been reported; hence, data regarding urgent surgical treatment of patients undergoing DBS presenting with ASDH are limited [7-11]. The results are presented in Table 1.

**Table 1:** Literature review of acute subdural hematoma with surgery in patients undergoing deep brain stimulation. NA, not available; M, male; F, female; L, left; DBS, deep brain stimulation; ASDH, acute subdural hematoma; PD, Parkinson’s disease; GCS, Glasgow Coma Scale; GOS, Glasgow Outcome Scale.

Case	Author/ Year	Age/Sex	Side	Period between DBS placement and ASDH	Cause of DBS	Cause of injury	GCS	Symptoms	Use of antithrombotic medication	Intervention for DBS system	Intervention for ASDH	Enlargement of ASDH	Length of hospitalization	GOS
1	Herzog J/ 2003 [11]	NA	NA	Intraoperative	PD	Ope	NA	NA	NA	Preserved	Surgical intervention	NA	NA	5
2	Kenney C/ 2007 [10]	NA	NA	Within 2 weeks	NA	NA	NA	NA	NA	Preserved	Evacuation	NA	NA	NA
3	Yang YJ/ 2013 [9]	67/ F	L	3 years	PD	Fall	3	Consciousness disturbance	NA	Preserved	Craniectomy	No	44 days	5
4	Nguyen HS/ 2015 [8]	68/ F	L	36 days	PD	NA	NA	Right hemiparesis	No	Preserved	Burr hole	No	6 days	5
5	Henderson EY/2015 [7]	57/M	L	1 week	PD	Fall	NA	Headache and confusion	No	Preserved	Craniotomy	Yes	NA	5
6	Present case	71/ F	L	8 years	PD	Unknown	7	Consciousness disturbance and right hemiparesis	No	Preserved	Small craniotomy with endoscopy	No	29 days	5

The management of SDH in patients undergoing DBS is extremely challenging because of the presence of heterogeneous treatment options, such as DBS function restriction, DBS system removal, hematoma removal, and a combination of these techniques. The first choice for ASDH treatment is a large craniotomy under general anesthesia. However, if the DBS system is removed, Parkinson’s disease cannot be treated; therefore, it must be reinserted later. Several surgeries and an increased burden of patient comorbidities may make invasive treatment strategies inappropriate.

Preservation of the DBS system

In a literature review, all DBS systems were preserved [7-11]. An SDH can cause the brain to shift because of increased pressure. Because the DBS electrodes are tethered to the skull, the ipsilateral electrode will be displaced from its original target site. In addition, due to the twisting of the electrodes, the patient may not respond well to stimulation. Significant electrode displacement raises the question of whether efforts should be made to preserve the electrodes in an emergency.

The gliosis formed along the track of the electrodes may function as a potential space; therefore, the migrated electrode may slide back to its original target site once the SDH is removed [4, 7-9].

In a literature review, the presence of ASDH after DBS also created a significant electrode displacement. After hematoma evacuation, the electrodes were successfully repositioned, and the patients demonstrated a favorable response to stimulation [7-11]. Therefore, DBS could remain effective without DBS revision surgery.

ASDH surgery for patients with DBS devices

Careful selection of surgical indications is necessary because compared with craniotomy or craniectomy for patients with ASDH, endoscopic surgery cannot decompress brain swelling. Therefore, our indications for endoscopic surgery for ASDH are as follows: presence of symptoms, absence of moderate or massive cerebral contusion/hematoma, and no high risk of bleeding. In addition, ICP monitoring allows for safe postoperative management and may improve outcomes.

Hematoma removal with preservation of the DBS system, such as burr hole, craniotomy, with/without endoscopy, and craniectomy, has been reported [7-11]. When a DBS system is in place, a large craniotomy involves a more significant surgical challenge than burr hole drainage. During a craniotomy, the DBS system should be avoided whenever feasible.

The number of reports on the use of endoscopic hematoma evacuation for ASDH is increasing [12-14]. Tanaka et al. reported that endoscopic hematoma evacuation of ASDH with a cerebrospinal fluid shunt is a safe and effective approach to preserve the shunt by making a skin incision parallel to the shunt catheter [14].

In our case, a skin incision parallel to the DBS lead during endoscopic small craniotomy hematoma evacuation preserved the DBS system. Subsequently, rapid recovery of neuropathy and restoration of DBS therapy were achieved in four weeks.

## Conclusions

ASDH in patients undergoing DBS causes a significant electrode displacement. After hematoma evacuation, the electrodes return to their optimal position and the patients exhibit a favorable clinical response to stimulation. To preserve the DBS system, it may be useful to perform endoscopic hematoma evacuation with a small craniotomy.
